# Impact of Transfer Learning on Convolutional Neural Networks for Odontogenic Tumor Diagnosis

**DOI:** 10.1007/s12105-025-01875-y

**Published:** 2026-02-19

**Authors:** Daniela Giraldo-Roldán, Thaís Cerqueira Reis Nakamura, Anderson Faria Claret, Giovanna Calabrese dos Santos, Katya Pulido-Díaz, Roberto Gerber-Mora, Leonor Victoria Gonzalez-Perez, Jeconias Câmara, Hélder Antônio Rebelo Pontes, Manoela Domingues Martin, Márcio Campos Oliveira, Vanesa Pereira Prado, Felipe Martins Silveira, Ronell Bologna-Molina, Anna Luíza Damaceno Araújo, Matheus Cardoso Moraes, Pablo Agustin Vargas

**Affiliations:** 1 Faculdade de Odontologia de Piracicaba, Department of Oral Diagnosis, Oral Pathology Area, Piracicaba Dental School, Universidade de Campinas (FOP- UNICAMP), Av. Limeira, 901, Piracicaba, São Paulo 13.414-903 Brazil; 2 Institute of Science and Technology, Federal University of São Paulo (ICT- Unifesp), São José dos Campos, São Paulo Brazil; 3 Health Care Department, Oral Pathology and Medicine Master, Autonomous Metropolitan University, Mexico City, Mexico; 4 Oroclinic, San José, Costa Rica; 5 Laboratory of Immunodetection and Bioanalysis, Investigation Group POPCAD, Faculty of Dentistry, University of Antioquia, Medellín, Colombia; 6 Department of Pathology and Legal Medicine, School of Medicine, Federal University of Amazonas, Manaus, Brazil; 7 Oral Pathology, João de Barros Barreto University Hospital, Federal University of Pará, Belem, Brazil; 8 Department of Oral Pathology, School of Dentistry, Federal University of Rio Grande do Sul, Porto Alegre, Brazil; 9 Department of Health, State University of Feira de Santana (UEFS), Feira de Santana, Brazil; 10 Department of Diagnostic Pathology and Oral Medicine, School of Dentistry, Universidad de la República (UDELAR), Montevideo, Uruguay; 11 Head and Neck Surgery Department, University of São Paulo Medical School, São Paulo, São Paulo Brazil; 12 Hospital Israelita Albert Einstein, São Paulo, Brazil

**Keywords:** Transfer learning, Convolutional neural networks, Automated diagnosis, Odontogenic tumors, Artificial intelligence, Deep learning

## Abstract

**Objective:**

This study aimed to evaluate the coherence between data heterogeneity and model complexity by comparing seven convolutional neural network (CNN) architectures—trained with and without ImageNet pretraining—in a multiclass framework for the histopathological classification of three odontogenic tumors: adenomatoid odontogenic tumor, ameloblastoma, and ameloblastic carcinoma. The goal was to investigate how transfer learning influences performance and diagnostic reliability in a clinically relevant context characterized by overlapping histological patterns.

**Methods:**

An international, multicenter cross-sectional dataset of 64 hematoxylin- and eosin-stained whole slide images was analyzed, including adenomatoid odontogenic tumor (*n* = 16), ameloblastoma (*n* = 27), and ameloblastic carcinoma (*n* = 21). Seven CNN models (DenseNet121, EfficientNetV2B0, InceptionV3, MobileNet, ResNet50, VGG16, and Xception) were trained and tested on 455,107 patches (224 × 224 pixels). Performance was assessed using accuracy, balanced accuracy, sensitivity, specificity, F1-score, and AUC.

**Results:**

Without ImageNet pretraining, DenseNet121 achieved the highest performance (accuracy = 0.73, balanced accuracy = 0.74, AUC = 0.78, specificity = 0.84, sensitivity = 0.65), followed by EfficientNetV2B0 (accuracy = 0.67, balanced accuracy = 0.68, sensitivity = 0.54). When ImageNet pretraining was applied, performance improved across all architectures. EfficientNetV2B0 reached the best overall results (accuracy = 0.79, balanced accuracy = 0.81, AUC = 0.91, specificity = 0.88, sensitivity = 0.74), while DenseNet121 maintained consistent performance (accuracy = 0.72, balanced accuracy = 0.74, AUC = 0.85, specificity = 0.84, sensitivity = 0.64).

**Conclusion:**

Transfer learning with ImageNet weights enhanced the performance of most CNNs, with EfficientNetV2B0 showing the greatest responsiveness to pretraining and DenseNet121 demonstrating intrinsic robustness to initialization. These results highlight the potential of CNN-based frameworks to support the differential diagnosis of odontogenic tumors—an inherently challenging task due to morphological overlap—while establishing reproducible methodological baselines that contribute to the global development of explainable, ensemble-based, and clinically reliable AI systems in oral pathology.

**Supplementary Information:**

The online version contains supplementary material available at 10.1007/s12105-025-01875-y.

## Introduction

Odontogenic tumors (OTs) are relatively rare lesions, accounting for up to 1% of oral and maxillofacial biopsies [1]. They comprise a heterogeneous group of lesions with a wide spectrum of clinical behavior, ranging from hamartomatous proliferations to malignant neoplasms with metastatic potential that, in aggressive cases, may become life-threatening [2]. The diagnosis of OTs is challenging due to their diverse clinical, radiographic, and microscopic presentations. Consequently, accurate diagnosis relies heavily on the expertise of oral and maxillofacial pathologists, whose interpretation of subtle histopathological features is essential for correct classification and treatment planning [3].

Histologically, odontogenic tumors often share overlapping features, such as the proliferation of odontogenic epithelium, which can make their differentiation difficult. This challenge becomes even greater in small or fragmented incisional biopsies, where limited tissue sampling may preclude the recognition of architectural patterns, resulting in descriptive or inconclusive reports. In such cases, the evaluation of the complete surgical specimen is frequently required for diagnostic confirmation [4]. Accurate differentiation among these entities is crucial, as their biological behavior and therapeutic implications differ considerably. While adenomatoid odontogenic tumor (AOT) is a benign and indolent lesion [5], ameloblastoma presents a locally invasive potential and occasional metastatic capacity [6], and ameloblastic carcinoma represents a true malignancy with aggressive evolution [7]. Therefore, establishing the correct diagnosis is essential to guide appropriate treatment, which ranges from conservative procedures to radical resections [8].

In this context, artificial intelligence (AI) has emerged as a promising tool to assist pathologists in the diagnostic interpretation of histopathological images. Advances in deep learning, particularly through convolutional neural networks (CNNs), have revolutionized medical image analysis by enabling the automatic extraction of complex visual patterns directly from raw data [9, 10]. In oral pathology, these models have demonstrated remarkable potential for lesion detection, classification, and segmentation tasks, achieving levels of accuracy and reproducibility that can approach or even surpass human performance in specific settings [11, 12]. Nevertheless, the performance of CNNs may vary substantially according to the heterogeneity of the dataset and the intrinsic complexity of each architecture, factors that remain underexplored in the context of odontogenic tumor classification.

Although deep learning approaches have been increasingly applied to oral and maxillofacial pathology, most existing studies have focused on binary classification tasks or on datasets with limited histological variability [13–15]. Few investigations have explored multiclass frameworks capable of distinguishing between histologically related entities such as AOT, ameloblastoma, and ameloblastic carcinoma. Moreover, literature lacks analyses addressing how the heterogeneity of histopathological data interacts with model complexity and transfer learning strategies. In the specific context of odontogenic tumors, this relationship remains largely unexplored, despite its relevance for understanding how CNN architectures adapt to varying sample diversity and for identifying models that achieve reliable generalization in clinical scenarios.

Given these considerations, the present study aimed to develop and evaluate a multiclass classification model based on convolutional neural networks (CNNs) to differentiate among AOT, ameloblastoma, and ameloblastic carcinoma, as well as to investigate the coherence between data variability and model complexity in the histopathological classification of odontogenic tumors. The influence of transfer learning on model performance and generalization was analyzed by comparing seven CNN architectures trained with and without ImageNet weights. This approach sought to identify the architectures that best balance accuracy and interpretability in a multiclass scenario, contributing to a reproducible framework for future AI-based diagnostic applications in oral pathology.

## Methods

### Image Acquisition and Data Collection

#### Sample

**Fig. 1:**
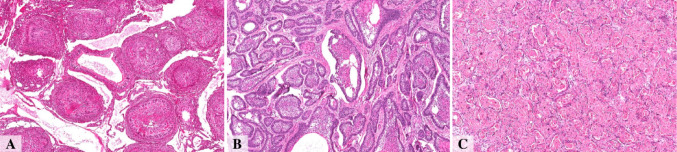
Representative histopathological features of adenomatoid odontogenic tumor (**A**), Ameloblastoma (**B**), and Ameloblastic Carcinoma (**C**). Displayed at 5×. Scale bar = 200 μm

A total of 64 cases diagnosed as adenomatoid odontogenic tumor (AOT, *n* = 16), ameloblastoma (*n* = 27), and ameloblastic carcinoma (*n* = 21) Fig. 1, supplementary appendix 1 were retrospectively collected from the archives of eight academic institutions in Latin America: the Department of Oral Pathology, School of Dentistry of Piracicaba (FOP-UNICAMP); the Department of Pathology and Legal Medicine, School of Medicine, Federal University of Amazonas (UFAM); the João de Barros Barreto University Hospital, Federal University of Pará (UFPA); the Department of Oral Pathology, School of Dentistry, Federal University of Rio Grande do Sul (UFRGS); the Department of Health, State University of Feira de Santana (UEFS); the Faculty of Dentistry, University of the Republic of Uruguay (UDELAR); the Faculty of Dentistry, Autonomous University of Mexico (UAM); and the Faculty of Dentistry, University of Antioquia (UdeA).

All diagnoses followed the current WHO Classification of Head and Neck Tumours, which allows the diagnosis of ameloblastic carcinoma based on H&E morphology when characteristic architectural and cytological atypia are present. Because specimens were obtained from multiple institutions, full resections and ancillary immunohistochemistry were not available for all cases; however, all diagnoses were confirmed by experienced oral and maxillofacial pathologists at the contributing centers (KPD; RGM; LVGP; JC; HARP; MDM; RBM; ALDA ; PAV).

Histological sections were stained with hematoxylin and eosin (H&E) and digitized into whole slide images (WSI) using the Aperio CS Digital Pathology System (Leica Biosystems, Wetzlar, Germany) at 20× magnification and a spatial resolution of 0.47 μm per pixel. Regions of interest (ROIs) corresponding to tumor parenchyma were manually identified by an experienced oral and maxillofacial pathologist (D.G.R.) and annotated in QuPath [16], an open-source platform for digital pathology image analysis. QuPath enables visualization at the cellular level, annotation of relevant tissue areas, and export of regions for deep learning applications. All annotations were independently reviewed by a second pathologist (K.P.D.) to ensure histopathological accuracy and consistency.

Following manual annotation, Computational processing was performed by the Biomedical Imaging and AI team under the supervision of Prof. Matheus Cardoso Moraes (M.C.M.), including T.C.R. Nakamura, A.F. Claret, and G.C. Santos.“. ROIs were segmented to retain only histopathologically relevant regions for model training. To meet the CNN input requirements, WSIs were divided into 224 × 224-pixel patches with a 30% overlap between adjacent tiles. Each patch was labeled according to the histopathological diagnosis of its source ROI: class 0 for AOT, class 1 for ameloblastoma, and class 2 for ameloblastic carcinoma. In total, 445,107 patches were obtained, distributed as follows: AOT (80,760), ameloblastoma (237,013), and ameloblastic carcinoma (127,334) (Fig. 3; Table 1).

**Fig. 2:**
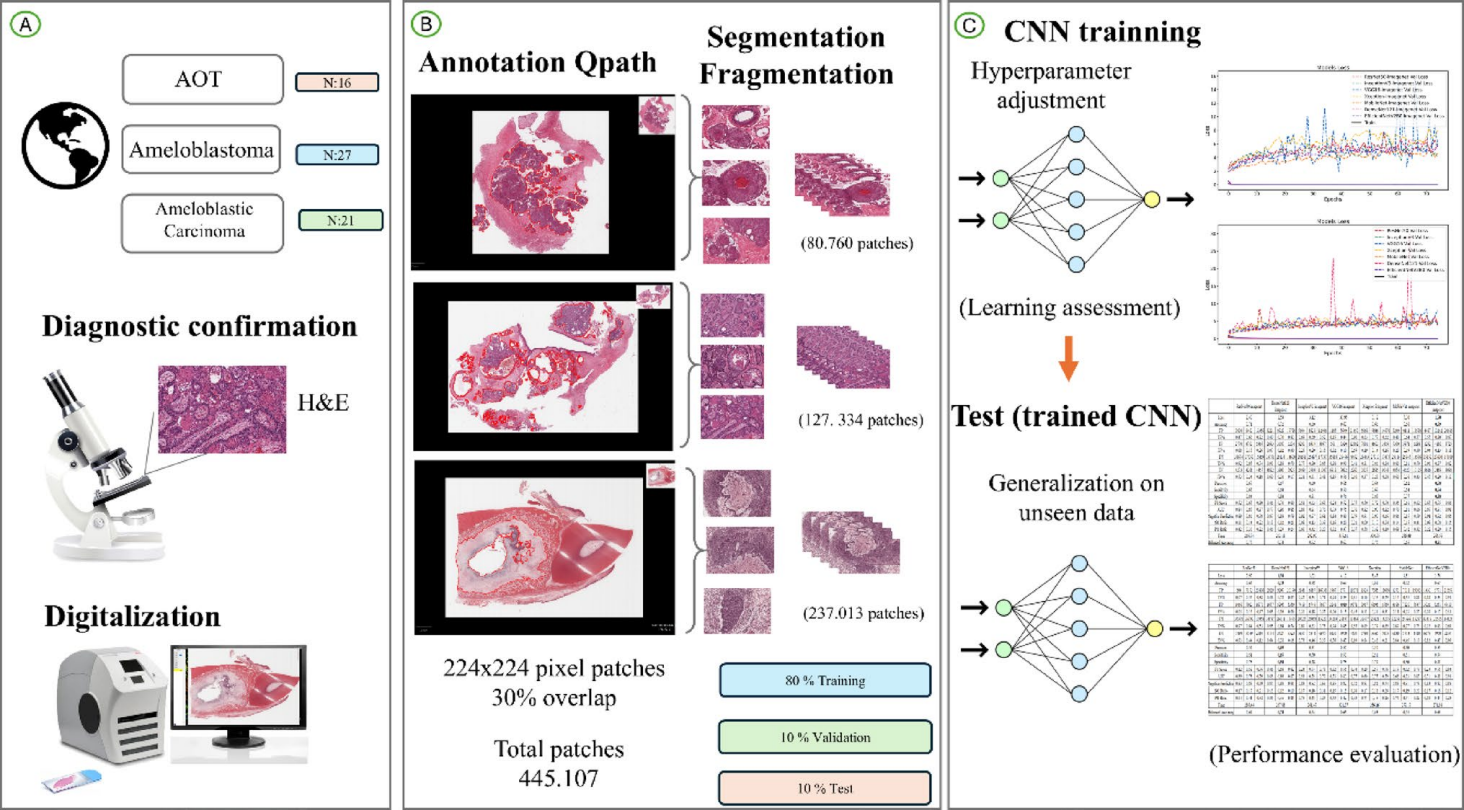
Methodological summary. **A** represents the initial stages of the process, including the acquisition of tissue samples and the digitization of the entire slide image (WSI). Panel (**B**) illustrates the annotation and classification of regions of interest (ROI), the subsequent segmentation and fragmentation of WSI into patches, and the division and distribution of patches into training, validation, and testing subsets, aiming for an 80:10:10 ratio. And panel (**C**) describes the evaluation process used for the performance of the proposed method, including the final performance illustrated through the loss function and the global metrics.

**Table 1:** Distribution of odontogenic tumor dataset

Class	0	1	2	Total
Slides	16	27	21	64
Segments	398	1394	1577	3369
Patches	80,760	127,334	237,013	445,107
Training	64,576	101,869	189,584	356,029
Validation	8081	12,734	23,716	44,531
Test	8103	12,731	23,713	44,547
Total	80,760	127,334	237,013	445,107

0 Adenomatoid odontogenic tumour; 1: ameloblastoma; 2: ameloblastic carcinoma

Patients were assigned to the training, validation, and test subsets using a patient-wise, non-random partitioning strategy with an 8:1:1 allocation ratio. This procedure ensured that all patches derived from a given case were confined to a single subset, thereby eliminating cross-patient data leakage and preserving the independence of the evaluation. Although the assignment was non-random, cases were distributed to approximate the proportional representation of each diagnostic category across subsets, minimizing class imbalance artifacts. This design preserved intra-class morphological heterogeneity within each split, an essential requirement for assessing the concordance between biological variability and model generalization capacity in the present study.

## CNN Architectures

Fourteen convolutional neural network (CNN) models were evaluated, comprising seven architectures trained from scratch and their corresponding counterparts initialized with ImageNet weights [17]. The selection included both classical and modern architectures to capture a wide range of structural complexities and connectivity patterns, enabling an assessment of how these factors interact with data heterogeneity.

The networks analyzed were ResNet50 [18], a residual architecture designed to mitigate vanishing gradients through skip connections and widely used as a robust benchmark for medical imaging tasks; DenseNet121 [19], characterized by dense inter-layer connections that promote feature reuse and efficient gradient propagation, offering strong performance even without pretraining; InceptionV3 [20], which incorporates multi-scale feature extraction through inception modules to optimize the capture of spatial details at multiple resolutions; VGG16 [21], a deep sequential model with uniform convolutional blocks, known for stability but containing a high number of parameters; Xception [22], based on depthwise separable convolutions and particularly effective in modeling complex textural and fine-grained histological features; MobileNet [23], a lightweight network designed for computational efficiency and real-time inference while maintaining reasonable accuracy with reduced parameters; and EfficientNetV2B0 [24], a compound-scaled model that balances depth, width, and resolution to achieve high accuracy with optimized resource use.

To ensure a fair comparison, identical hyperparameters were applied to all models: batch size of 32, Adam optimizer with a learning rate of 1 × 10⁻⁵, and categorical cross-entropy loss function. Each model was trained for 75 epochs. In addition to training from scratch, a transfer learning strategy was employed using ImageNet-pretrained weights from the ILSVRC dataset [17], allowing analysis of how prior feature representations influence learning dynamics and generalization in a histopathological multiclass scenario.

## Hardware

**Fig. 3:**
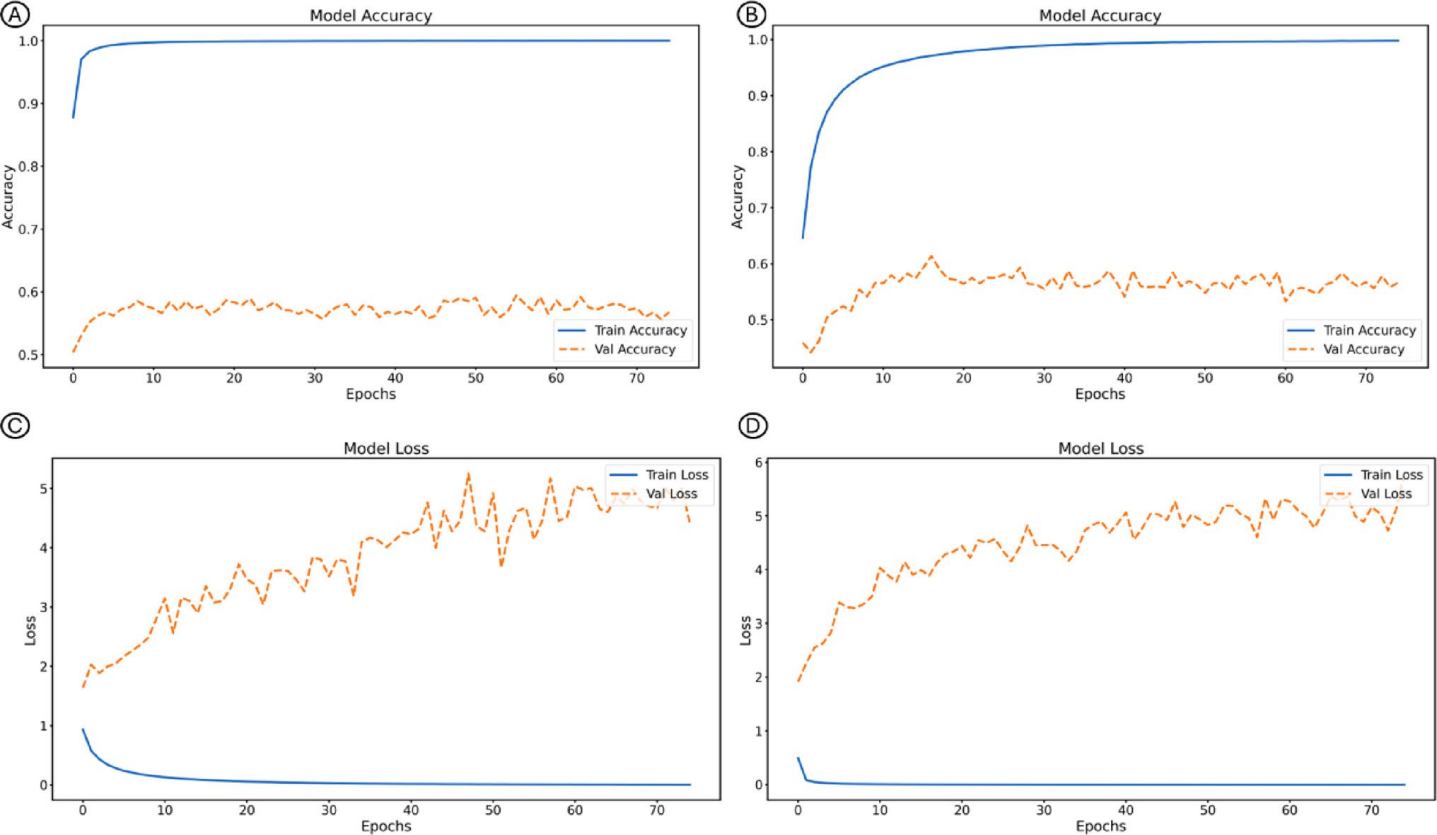
Accuracy and loss curves of the EfficientNetV2B0 model during training and validation, comparing the strategy without pretraining (**A**) and (**B**), and with pretraining in ImageNet (**C**) and (**D**)

All experiments were conducted on a workstation equipped with a 13th Gen Intel^®^ Core™ i7-13700KF 3.40 GHz processor, 128 GB of RAM, and running the Windows 11 Pro 64-bit operating system. Model development and analysis were performed using Spyder IDE 5.5.1 and Python 3.10.14. A dedicated NVIDIA GeForce RTX 4090 GPU with 24 GB of memory was employed to accelerate training and inference processes. The convolutional neural networks were implemented using TensorFlow 2.10.0, Keras 2.10.0, scikit-plot 0.3.7, and Matplotlib 3.10.1. The complete pipeline followed the methodological workflow summarized in Fig. 3, ensuring reproducibility and consistency across all experimental stages.

## Evaluation

To evaluate the learning capacity and generalization of the models, accuracy and loss curves were generated and compared across both training and validation datasets. Generalization performance was further assessed through a comprehensive set of quantitative metrics obtained during the testing phase, enabling a detailed analysis of each model’s predictive behavior. The metrics, summarized in Supplementary Appendix 2 and 3, included loss, accuracy, balanced accuracy, precision, sensitivity (recall), specificity, F1-score, area under the receiver operating characteristic curve (AUC), and the corresponding counts of true positives (TP), true negatives (TN), false positives (FP), and false negatives (FN) [19, 24].

Additionally, computational efficiency was evaluated by measuring inference time during the testing phase, providing insight into each architecture’s practical feasibility for clinical implementation. Confidence intervals were not computed because the dataset operates at the patch level and does not represent statistically independent sampling units. Traditional resampling strategies, such as k-fold cross-validation or bootstrap, were considered inappropriate for this context, as they would either lead to data leakage between patient samples or fail to provide meaningful variance estimates without independent experimental repetitions. Instead, patient-wise data split was adopted to preserve biological heterogeneity and ensure that performance metrics reflected model robustness rather than data redundancy.

## Results

The final performance metrics for the fourteen convolutional neural network (CNN) configurations comprising seven architectures trained with and without ImageNet pretraining. Overall, the results demonstrate that deep learning models can reliably perform multiclass classification of odontogenic tumors classification of odontogenic tumors, distinguishing adenomatoid odontogenic tumor (AOT), ameloblastoma, and ameloblastic carcinoma with clinically relevant accuracy. The performance trends observed across architectures demonstrate consistent improvements with transfer learning, confirming the feasibility of applying CNN-based methods to complex histopathological datasets. (Supplementary Appendix 4).

**Fig. 4:**
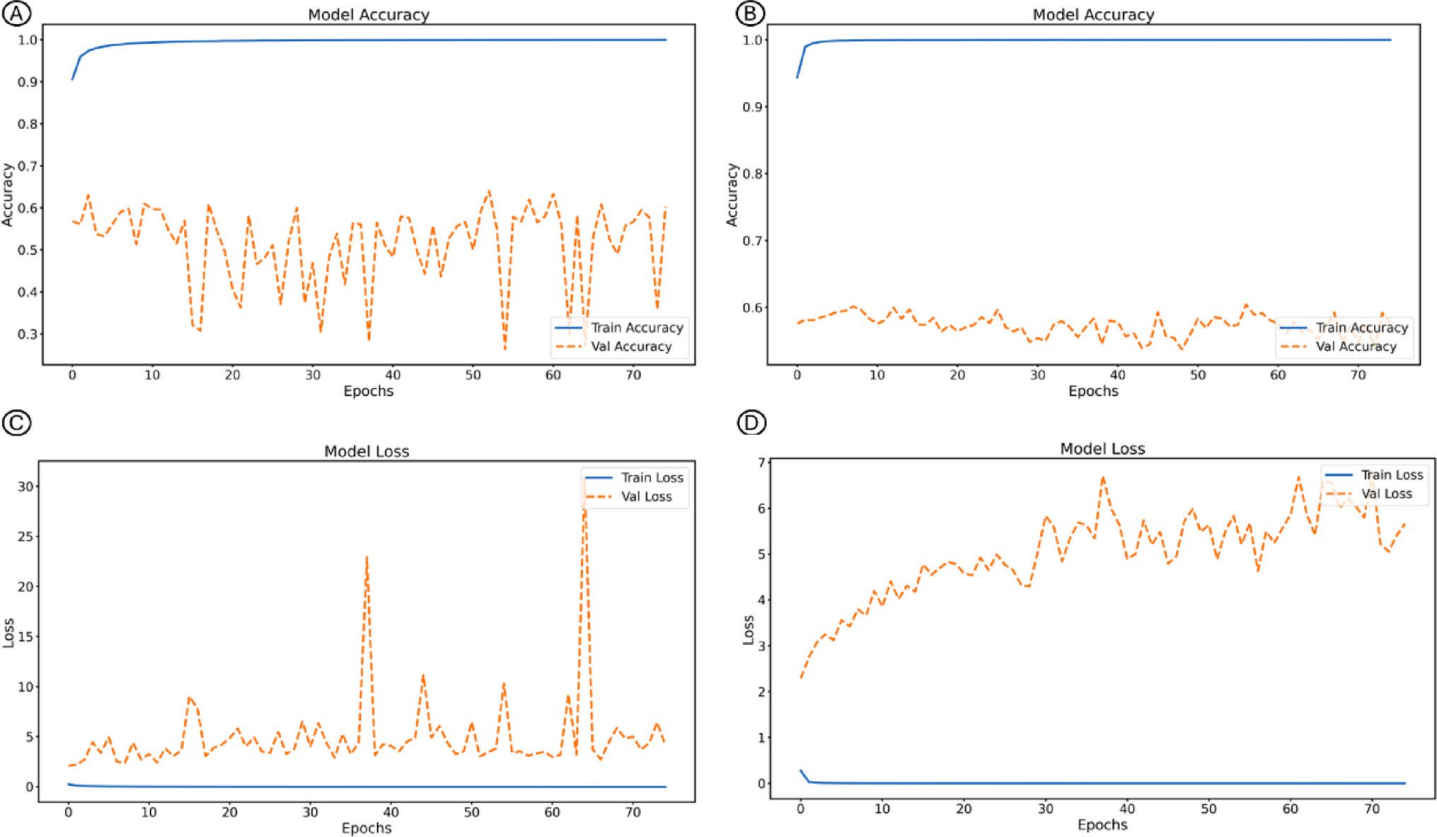
Accuracy and loss curves of the DenseNet-121 model during training and validation, comparing the strategy without pretraining (**A**) and (**B**), and with pretraining in ImageNet (**C**) and (**D**)

When evaluated with ImageNet pretraining, the overall best performance was obtained with EfficientNetV2B0, which reached an accuracy of 0.79, a balanced accuracy of 0.81, an AUC of 0.91, a specificity of 0.88, and a sensitivity of 0.74. This architecture also presented the lowest loss (1.70), followed by DenseNet121, which achieved an accuracy of 0.72, a balanced accuracy of 0.74, and an AUC of 0.90, demonstrating a solid balance between sensitivity and specificity. In contrast, VGG16 and MobileNet showed weaker performance, with balanced accuracies of 0.63 and 0.65, respectively, and a considerably higher loss for VGG16 (33.95). These findings confirm that pre-trained weights substantially improve model performance, with EfficientNetV2B0 standing out as the best overall architecture under these conditions (Fig. 4).

Among the CNNs trained from scratch (without ImageNet pretraining), DenseNet121 achieved the best overall performance, with an accuracy of 0.73, a balanced accuracy of 0.74, an AUC of 0.86, a specificity of 0.84, and a sensitivity of 0.65. It also exhibited the lowest loss (1.94), indicating efficient convergence and strong generalization even without prior weights. EfficientNetV2B0 also performed consistently well but showed a reduction in sensitivity (0.54) compared with DenseNet121. ResNet50 and InceptionV3 presented comparable results, both achieving a balanced accuracy of 0.64. These findings suggest that DenseNet121 was the most robust network when trained exclusively on domain-specific histological data (Fig. 3).

When comparing both training strategies, ImageNet pretraining improved performance across all architectures, except for DenseNet121, whose results slightly declined after transfer learning. EfficientNetV2B0 showed the greatest performance gain with pretraining, achieving the highest accuracy and sensitivity among all models. MobileNet remained the fastest architecture (255 s with ImageNet and 256 s without), although it sacrificed some accuracy. EfficientNetV2B0 achieved an excellent balance between moderate inference time (~ 265 s) and high predictive performance. Overall, EfficientNetV2B0 and DenseNet121 stood out as the most reliable models for multiclass classification of odontogenic tumors, each excelling under different training conditions.

An additional analysis focused on the coherence between sample heterogeneity and model performance. In this context, the AOT class (class 0), which comprised fewer and more histologically homogeneous samples (80,760 patches from 16 cases), exhibited greater variability in classification accuracy across models. This behavior suggests that limited intra-class variability may have restricted the ability of the networks to generalize discriminative features effectively. Rather than representing a limitation, this finding reinforces the study’s objective of examining how differences in sample diversity interact with network complexity to influence learning stability. The histopathological spectrum of AOT may require additional representative cases—either original or synthetic (GAN-generated)—or the implementation of ensemble-based architectures capable of compensating for reduced heterogeneity and enhancing generalization in future classification models.

## Discussion

Images constitute the fundamental input in any computer vision–based classification project, particularly in medical and histopathological applications [9–11, 25]. Each image encodes morphological information that a convolutional neural network (CNN) can learn to recognize, classify, or segment—such as cellular patterns, tissue organization, textures, and color variations [9, 10]. In histopathology, the use of digitized slides enables CNNs to distinguish tumor types according to their visual characteristics, emulating the diagnostic reasoning process of pathologists but on a larger and faster scale [10, 26]. The quality, quantity, annotation accuracy, and preprocessing of these images directly influence model performance [27, 28]. Data augmentation, for instance, remains an essential strategy to simulate the natural variability observed in clinical settings and to increase robustness against artifacts or staining variations [29, 30].

ImageNet [17] is a large-scale database containing millions of labeled natural images from thousands of categories. For more than a decade, it has been widely used as a reference dataset for training deep neural networks in visual recognition tasks [31]. Models pre-trained on ImageNet acquire a broad representation of the visual world, learning to recognize generic patterns such as edges, textures, and spatial structures. When these pre-trained weights are transferred to new domains, the network does not start from random initialization but from a set of generalized visual features already optimized for pattern recognition [31]. This strategy, known as transfer learning, offers several advantages: the initial layers of a CNN capture universal visual features that can be reused for specialized domains such as medical imaging [32], allowing efficient learning even when the available dataset is relatively small. Pre-trained models usually converge faster, achieve higher accuracy, and generalize better, acting as an implicit regularization mechanism that reduces overfitting [32, 33].

In the present study, the images represented real clinical cases of odontogenic tumors, and the networks pre-trained on ImageNet allowed the models to leverage prior visual knowledge to improve classification performance across three histological entities: AOT, ameloblastoma, and ameloblastic carcinoma. This approach is particularly relevant given that these lesions belong to a morphological continuum—from benign to malignant—where the visual differences between classes can be subtle.

A clearer justification of the clinical value of this study involves recognizing that differentiating AOT from ameloblastoma is generally straightforward for most experienced pathologists when representative tissue sections are available. However, this does not diminish the interest in evaluating how AI behaves when classifying AOT, since non-representative, fragmented, or poorly preserved biopsies may occasionally obscure classic features. More importantly, the true diagnostic challenge lies in distinguishing ameloblastoma from ameloblastic carcinoma, an area characterized by overlapping architectural features, variable cytologic atypia, and documented interobserver variability—including findings already reported by our group [14]. AI-based tools, particularly CNN-driven classification and future AI-assisted segmentation, hold potential as objective adjuncts in these complex scenarios by consistently highlighting architectural and cytological cues that may be subtle or inconsistently appreciated.

The clinical utility of AI becomes even more apparent when considering rarer odontogenic entities such as adenoid ameloblastoma, primordial odontogenic tumor, and dentinogenic ghost cell tumor—lesions that are infrequently encountered in routine practice and may not be readily recognized by general pathologists. In this context, the development and benchmarking of AI models contribute not only to supporting difficult differential diagnoses but also to expanding diagnostic reliability in uncommon tumors where familiarity is limited. This perspective aligns with the broader goal of enhancing diagnostic consistency through computational tools while acknowledging that the models remain experimental and require further validation before clinical integration.

Transfer learning thus plays a critical role in enhancing feature discrimination, enabling CNNs to capture architectural and cytological nuances that may escape manual interpretation. Such behavior is consistent with previous studies showing that ImageNet pretraining enhances learning efficiency and diagnostic precision in medical imaging tasks involving limited and heterogeneous datasets [13, 14, 34–36].

The application of artificial intelligence in histological image classification has grown exponentially, largely due to the transformative impact of convolutional neural networks on medical image analysis [9, 25]. These models can identify microscopic patterns that are often imperceptible to the human eye, such as variations in epithelial organization, nuclear morphology, and stromal characteristics [14, 34–36]. In the context of odontogenic tumors, CNNs can automatically analyze regions of interest (ROIs) from digitized slides, detecting subtle differences in epithelial architecture, cell arrangements, and stromal textures that are essential for distinguishing among histological subtypes. This capability not only emulates but can also augment the diagnostic reasoning process of oral and maxillofacial pathologists, allowing large-scale, reproducible, and time-efficient analyses that support clinical decision-making.

Only a few studies have implemented CNNs using histological slides for the diagnosis of odontogenic lesions, and most of them have focused on benign or inflammatory cysts rather than true neoplasms. Florindo et al. [37] applied Bouligand–Minkowski fractal descriptors combined with machine learning to differentiate odontogenic keratocysts (OKCs) from radicular cysts, achieving 98% discrimination accuracy for OKCs versus radicular cysts and 68% for OKC subtypes. More recently, Cai et al. [13] developed an InceptionV3-based model for whole-slide image classification and prognosis of OKCs, distinguishing syndromic from non-syndromic lesions with diagnostic and prognostic AUCs of 0.935 and 0.840, respectively. Similarly, Rao et al. [38] used VGG16 and DenseNet169 to classify cystic lesions, reporting 93% accuracy in OKC versus non-OKC discrimination. Among the few studies addressing neoplastic entities, Giraldo-Roldán et al. [14] classified ameloblastomas and ameloblastic carcinomas using ResNet50, DenseNet, and VGG16, reaching 0.98 accuracy and 0.98 AUC. Additionally, Kim et al. [39] investigated the concordance between clinical and histopathological diagnoses of oral lesions using ChatGPT-4 and a Bayesian model (ORAD), obtaining 41.4% and 45.6% diagnostic concordance, respectively.

Taken together, these studies highlight the growing potential of deep learning in oral pathology but also reveal a gap: most investigations are limited to binary or small-class problems. The present study expands this field by addressing a more challenging multiclass scenario and by analyzing how data variability and model complexity interact under transfer learning, providing methodological evidence for future diagnostic frameworks in oral and maxillofacial pathology.

The ability of deep learning models to achieve multiclass diagnosis is crucial for this clinical application, as odontogenic tumors often share overlapping histological patterns such as palisaded epithelial arrangements, cellular nests, and calcification foci [2, 40]. However, subtle but diagnostically significant differences—such as the degree of cellular atypia, nuclear polarity, and stromal invasion—can be quantitatively captured by CNNs trained on high-quality, well-annotated datasets. Preprocessing strategies such as color normalization and class balancing help mitigate biases caused by staining variability and unequal sample sizes [27, 28], while data augmentation and transfer learning further enhance generalization and prevent overfitting, particularly in rare pathologies with limited case numbers [30]. In this context, the approach adopted in this study demonstrates that even with heterogeneous sample distributions, model robustness can be achieved through a combination of architectural optimization and careful dataset preparation.

The clinical implications of applying artificial intelligence to the diagnosis of odontogenic tumors are substantial. By reducing subjectivity and interobserver variability, AI-assisted systems can increase diagnostic consistency and confidence, particularly in borderline or ambiguous cases. Moreover, the ability to rapidly process large volumes of histological data enables pathologists to focus their expertise on complex or uncertain diagnoses, while automated models handle more routine classifications. Such approaches also hold promise for use in resource-limited settings, where access to specialized oral pathologists is often scarce, thereby contributing to the democratization of accurate and timely diagnosis.

The findings of this study illustrate how artificial intelligence can complement the expertise of oral and maxillofacial pathologists, helping to overcome human limitations in visual interpretation, reduce diagnostic time, and support more precise and patient-centered decision-making. Beyond quantifying model performance, this work represents an incremental step in the ongoing development of AI-based diagnostic frameworks for oral pathology. By documenting and validating methodological advances, it contributes to the accumulation of reproducible knowledge that can guide subsequent studies and improve future diagnostic architectures. Such continuity is essential to sustain collaborative, data-driven progress toward clinically reliable and explainable systems, integrating ensemble strategies, multimodal approaches, and interpretability tools as key components of the next generation of medical AI.

The study is limited by the cohort size (*n* = 64) due to tumor rarity; however, this was mitigated by a large-scale dataset of 445,107 patches using strict patient-wise splitting to provide variability for feature learning. Although scanned at 20×, accuracy was preserved through high-zoom digital annotation. Class imbalance was addressed via balanced metrics. While full resections and IHC were not available for all cases, diagnoses strictly followed WHO criteria confirmed by expert pathologists. Finally, as the study focused on quantitative architectural benchmarking, qualitative interpretability analyses (e.g., Grad-CAM) were outside the current scope and are reserved for dedicated future investigation.

Although the models demonstrated promising diagnostic performance, this study remains experimental. The approach has not yet been tested in clinical workflows, prospective settings, or external multi-institutional deployments. Therefore, the findings should not be interpreted as evidence of clinical readiness. Instead, they support the feasibility of applying CNN-based methods to odontogenic tumor classification and highlight methodological considerations for future development.

## Conclusions

This study demonstrates the strong potential of convolutional neural networks, combined with transfer learning strategies, for the multiclass classification of odontogenic tumors using histological slides. Among the evaluated architectures, DenseNet121 and EfficientNetV2B0 achieved the best performances, highlighting the ability of deep learning models to distinguish between lesions that share complex histopathological features. By integrating the accuracy of CNNs with optimization approaches adapted to the visual and morphological characteristics of oral pathology, this framework has the potential to transform diagnostic workflows—improving both quality and efficiency in the clinical management of these tumors.

The findings of this work illustrate how artificial intelligence can complement the expertise of oral and maxillofacial pathologists, reducing diagnostic subjectivity, accelerating analysis time, and supporting more consistent, patient-centered decision-making. Beyond reporting model performance, this research represents an incremental step in the evolution of AI-based diagnostic systems, documenting methodological progress that can be reproduced, compared, and expanded by future investigations.

Furthermore, this study aligns with the ongoing worldwide effort to build cumulative knowledge and establish methodological checkpoints toward the creation of complex, efficient, and trustworthy diagnostic systems. By documenting and sharing incremental advances, it contributes to the collective scientific foundation required for globally interoperable and clinically reliable AI tools. Given the growing interest and evidence supporting AI performance in medicine, continued efforts toward clinical validation, interpretability, and ethical integration are essential to ensure safe and meaningful translation into routine practice.

### Future Guidelines

While this study focused on the three most commonly encountered odontogenic tumors, rarer entities such as adenoid ameloblastoma and primordial odontogenic tumor represent important future directions. Additionally, future work may explore GAN-generated samples or ensemble approaches to address class imbalance in these rarer subtypes.

The differences in performance observed among the architectures may be related to their structural depth, number of parameters, and distinct feature extraction mechanisms. Future studies should explore each model in greater detail to maximize their strengths and develop complementary ensemble approaches that combine their advantages. In this context, balancing diagnostic accuracy with inference time will be crucial to ensure clinical feasibility and real-world applicability.

Expanding the dataset to include larger and more diverse samples remains a key priority for reducing overfitting and improving model robustness. Strategies aimed at enhancing generalization—such as systematic hyperparameter tuning, neural architecture search (NAS), and optimized pooling or kernel configurations—should be investigated to identify optimal performance trade-offs. Moreover, integrating multimodal data, including clinical and radiographic information alongside histological images, could provide a more holistic representation of patient cases, better reflecting the complexity of diagnostic workflows in practice.

Finally, improving interpretability is essential for clinical translation. Future frameworks should incorporate explainable AI (XAI) techniques capable of elucidating decision-making processes and identifying histological patterns that drive predictions. These efforts, together with open data sharing and transparent benchmarking, will strengthen the reproducibility and ethical development of AI systems in pathology—advancing the global endeavor toward reliable, explainable, and equitable diagnostic intelligence.

## Supplementary Information

Below is the link to the electronic supplementary material.

Supplementary Material 1

Supplementary Material 2

Supplementary Material 3

Supplementary Material 4

## Data Availability

Raw data were generated at [Piracicaba School of Dentistry, University of Campinas (FOP-UNICAMP), and Institute of Science and Technology, Federal University of São Paulo]. Derived data supporting the findings of this study are available from the corresponding author [PAV] on request.
