# Supplementary material for: Impact of Transfer Learning on Convolutional Neural Networks for Odontogenic Tumor Diagnosis
Source: Head Neck Pathol. 2026 Feb 19;20(1):24. doi: 10.1007/s12105-025-01875-y (PMC12920825; doi:10.1007/s12105-025-01875-y)
Supplement: Supplementary file 1 — Supplementary Material 1 [file 12105_2025_1875_MOESM1_ESM.docx]

**Supplementary Appendix 1.** Evaluation metrics for classification of odontogenic tumors without pre-trained weights

| **Models** | **ResNet50** | | | **DenseNet121** | | | **InceptionV3** | | | **VGG16** | | | **Xception** | | | **MobileNet** | | | **EfficientNetV2B0** | | |
| --- | --- | --- | --- | --- | --- | --- | --- | --- | --- | --- | --- | --- | --- | --- | --- | --- | --- | --- | --- | --- | --- |
| **Classes** | 0 | 1 | 2 | 0 | 1 | 2 | 0 | 1 | 2 | 0 | 1 | 2 | 0 | 1 | 2 | 0 | 1 | 2 | 0 | 1 | 2 |
| **TP** | 604 | 7172 | 20806 | 2929 | 9207 | 20190 | 2148 | 6887 | 16738 | 1967 | 6771 | 19278 | 1323 | 7365 | 18638 | 1273 | 7018 | 19303 | 604 | 7172 | 20806 |
| **TP%** | 0,07 | 0,56 | 0,88 | 0,36 | 0,72 | 0,85 | 0,27 | 0,54 | 0,71 | 0,24 | 0,53 | 0,81 | 0,16 | 0,58 | 0,79 | 0,16 | 0,55 | 0,81 | 0,07 | 0,56 | 0,88 |
| **FP** | 1066 | 5023 | 9876 | 1657 | 5205 | 5359 | 7419 | 5748 | 5607 | 2241 | 4919 | 9371 | 3967 | 6695 | 6559 | 4186 | 7220 | 5547 | 1066 | 5023 | 9876 |
| **FP%** | 0,03 | 0,16 | 0,47 | 0,05 | 0,16 | 0,26 | 0,20 | 0,18 | 0,27 | 0,06 | 0,15 | 0,45 | 0,11 | 0,21 | 0,31 | 0,11 | 0,23 | 0,27 | 0,03 | 0,16 | 0,47 |
| **TN** | 35378 | 26793 | 10958 | 34787 | 26611 | 15475 | 29025 | 26068 | 15227 | 34203 | 26897 | 11463 | 32477 | 25121 | 14275 | 32258 | 24596 | 15287 | 35378 | 26793 | 10958 |
| **TN%** | 0,97 | 0,84 | 0,53 | 0,95 | 0,84 | 0,74 | 0,80 | 0,82 | 0,73 | 0,94 | 0,85 | 0,55 | 0,89 | 0,79 | 0,69 | 0,89 | 0,77 | 0,73 | 0,97 | 0,84 | 0,53 |
| **FN** | 7499 | 5559 | 2907 | 5174 | 3524 | 3523 | 5955 | 5844 | 6975 | 6136 | 5960 | 4435 | 6780 | 5366 | 5075 | 6830 | 5713 | 4410 | 7499 | 5559 | 2907 |
| **FN%** | 0,93 | 0,44 | 0,12 | 0,64 | 0,28 | 0,15 | 0,73 | 0,46 | 0,29 | 0,76 | 0,47 | 0,19 | 0,84 | 0,42 | 0,21 | 0,84 | 0,45 | 0,19 | 0,93 | 0,44 | 0,12 |
| **F1 Score** | 0,12 | 0,58 | 0,76 | 0,46 | 0,68 | 0,82 | 0,24 | 0,54 | 0,73 | 0,32 | 0,55 | 0,74 | 0,20 | 0,55 | 0,76 | 0,19 | 0,52 | 0,79 | 0,23 | 0,55 | 0,84 |
| **AUC** | 0,69 | 0,78 | 0,79 | 0,83 | 0,86 | 0,87 | 0,58 | 0,75 | 0,79 | 0,73 | 0,80 | 0,77 | 0,60 | 0,77 | 0,79 | 0,66 | 0,73 | 0,87 | 0,71 | 0,81 | 0,91 |
| **Negative prediction value** | 0,83 | 0,83 | 0,79 | 0,87 | 0,88 | 0,81 | 0,83 | 0,82 | 0,69 | 0,85 | 0,82 | 0,72 | 0,83 | 0,82 | 0,74 | 0,83 | 0,81 | 0,78 | 0,83 | 0,83 | 0,79 |
| **NO ratio** | 0,17 | 0,17 | 0,21 | 0,13 | 0,12 | 0,19 | 0,17 | 0,18 | 0,31 | 0,15 | 0,18 | 0,28 | 0,17 | 0,18 | 0,26 | 0,17 | 0,19 | 0,22 | 0,17 | 0,17 | 0,21 |
| **PO ratio** | 0,64 | 0,41 | 0,32 | 0,36 | 0,36 | 0,21 | 0,78 | 0,45 | 0,25 | 0,53 | 0,42 | 0,33 | 0,75 | 0,48 | 0,26 | 0,77 | 0,51 | 0,22 | 0,64 | 0,41 | 0,32 |
| **Loss** | 2,99 | | | **1,94** | | | 3,02 | | | 4,12 | | | 3,47 | | | 3,51 | | | 2,76 | | |
| **Accuracy** | 0,64 | | | **0,73** | | | 0,58 | | | 0,63 | | | 0,61 | | | 0,62 | | | 0,67 | | |
| **Balanced accuracy** | 0,64 | | | **0,74** | | | 0,64 | | | 0,65 | | | 0,65 | | | 0,65 | | | 0,68 | | |
| **Precision** | 0,54 | | | **0,69** | | | 0,51 | | | 0,57 | | | 0,50 | | | 0,50 | | | 0,55 | | |
| **Sensitivity** | 0,51 | | | **0,65** | | | 0,50 | | | 0,53 | | | 0,51 | | | 0,51 | | | 0,54 | | |
| **Specificity** | 0,78 | | | **0,84** | | | 0,78 | | | 0,78 | | | 0,79 | | | 0,80 | | | 0,81 | | |
| **Time** | 286,44 | | | 267,88 | | | 261,47 | | | 321,37 | | | **256,16** | | | 259,19 | | | 271,88 | | |

TP: True Positive; FP: False Positive; TN: True Negative; FN: False Negative; AUC: Area Under the Curve. 0: adenomatoid odontogenic tumour; 1: ameloblastoma; 2: ameloblastic carcinoma.
