# Supplementary material for: Impact of Transfer Learning on Convolutional Neural Networks for Odontogenic Tumor Diagnosis
Source: Head Neck Pathol. 2026 Feb 19;20(1):24. doi: 10.1007/s12105-025-01875-y (PMC12920825; doi:10.1007/s12105-025-01875-y)
Supplement: Supplementary file 2 — Supplementary Material 2 [file 12105_2025_1875_MOESM2_ESM.docx]

**Supplementary Appendix 2**. Evaluation metrics for classification of odontogenic tumors with pre-trained weights (ImageNet).

| **Models** | **ResNet50** | | | **DenseNet121** | | | **InceptionV3** | | | **VGG16** | | | **Xception** | | | **MobileNet** | | | **EfficientNetV2B0** | | |
| --- | --- | --- | --- | --- | --- | --- | --- | --- | --- | --- | --- | --- | --- | --- | --- | --- | --- | --- | --- | --- | --- |
| **Classes** | 0 | 1 | 2 | 0 | 1 | 2 | 0 | 1 | 2 | 0 | 1 | 2 | 0 | 1 | 2 | 0 | 1 | 2 | 0 | 1 | 2 |
| **TP** | 3830 | 8463 | 19356 | 3221 | 8928 | 19788 | 5604 | 8828 | 12406 | 1185 | 5649 | 21450 | 5068 | 9846 | 14670 | 3249 | 8111 | 13588 | 4487 | 10242 | 20615 |
| **TP%** | 0,47 | 0,66 | 0,82 | 0,40 | 0,70 | 0,83 | 0,69 | 0,69 | 0,52 | 0,15 | 0,44 | 0,90 | 0,63 | 0,77 | 0,62 | 0,40 | 0,64 | 0,57 | 0,55 | 0,80 | 0,87 |
| **FP** | 2770 | 4763 | 5365 | 2693 | 3683 | 6234 | 8293 | 6319 | 3097 | 561 | 3320 | 12382 | 7001 | 4603 | 3359 | 7330 | 5971 | 6298 | 1292 | 4186 | 3725 |
| **FP%** | 0,08 | 0,15 | 0,26 | 0,07 | 0,12 | 0,30 | 0,23 | 0,20 | 0,15 | 0,02 | 0,10 | 0,59 | 0,19 | 0,14 | 0,16 | 0,20 | 0,19 | 0,30 | 0,04 | 0,13 | 0,18 |
| **TN** | 33674 | 27053 | 15469 | 33751 | 28133 | 14600 | 28151 | 25497 | 17737 | 35883 | 28496 | 8452 | 29443 | 27213 | 17475 | 29114 | 25845 | 14536 | 35152 | 27630 | 17109 |
| **TN%** | 0,92 | 0,85 | 0,74 | 0,93 | 0,88 | 0,70 | 0,77 | 0,80 | 0,85 | 0,98 | 0,90 | 0,41 | 0,81 | 0,86 | 0,84 | 0,80 | 0,81 | 0,70 | 0,96 | 0,87 | 0,82 |
| **FN** | 4273 | 4268 | 4357 | 4882 | 3803 | 3925 | 2499 | 3903 | 11307 | 6918 | 7082 | 2263 | 3035 | 2885 | 9043 | 4854 | 4620 | 10125 | 3616 | 2489 | 3098 |
| **FN%** | 0,53 | 0,34 | 0,18 | 0,60 | 0,30 | 0,17 | 0,31 | 0,31 | 0,48 | 0,85 | 0,56 | 0,10 | 0,37 | 0,23 | 0,38 | 0,60 | 0,36 | 0,43 | 0,45 | 0,20 | 0,13 |
| **F1 Score** | 0,52 | 0,65 | 0,80 | 0,46 | 0,70 | 0,80 | 0,51 | 0,63 | 0,63 | 0,24 | 0,52 | 0,75 | 0,50 | 0,72 | 0,70 | 0,35 | 0,61 | 0,62 | 0,65 | 0,75 | 0,86 |
| **AUC** | 0,84 | 0,85 | 0,87 | 0,77 | 0,90 | 0,85 | 0,83 | 0,81 | 0,76 | 0,73 | 0,75 | 0,76 | 0,82 | 0,90 | 0,82 | 0,70 | 0,81 | 0,66 | 0,87 | 0,91 | 0,91 |
| **Negative prediction value** | 0,89 | 0,86 | 0,78 | 0,87 | 0,88 | 0,79 | 0,92 | 0,87 | 0,61 | 0,84 | 0,80 | 0,79 | 0,91 | 0,90 | 0,66 | 0,86 | 0,85 | 0,59 | 0,91 | 0,92 | 0,85 |
| **NO ratio** | 0,11 | 0,14 | 0,22 | 0,13 | 0,12 | 0,21 | 0,08 | 0,13 | 0,39 | 0,16 | 0,20 | 0,21 | 0,09 | 0,10 | 0,34 | 0,14 | 0,15 | 0,41 | 0,09 | 0,08 | 0,15 |
| **PO ratio** | 0,42 | 0,36 | 0,22 | 0,46 | 0,29 | 0,24 | 0,60 | 0,42 | 0,20 | 0,32 | 0,37 | 0,37 | 0,58 | 0,32 | 0,19 | 0,69 | 0,42 | 0,32 | 0,22 | 0,29 | 0,15 |
| **Loss** | 2,40 | | | 2,53 | | | 3,12 | | | 33,95 | | | 3,12 | | | 5,30 | | | **1,70** | | |
| **Accuracy** | 0,71 | | | 0,72 | | | 0,60 | | | 0,63 | | | 0,66 | | | 0,56 | | | **0,79** | | |
| **Balanced accuracy** | 0,75 | | | 0,74 | | | 0,72 | | | 0,63 | | | 0,75 | | | 0,65 | | | **0,81** | | |
| **Precision** | 0,67 | | | 0,67 | | | 0,60 | | | 0,65 | | | 0,64 | | | 0,52 | | | **0,78** | | |
| **Sensitivity** | 0,65 | | | 0,64 | | | 0,64 | | | 0,50 | | | 0,67 | | | 0,54 | | | **0,74** | | |
| **Specificity** | 0,84 | | | 0,84 | | | 0,81 | | | 0,76 | | | 0,83 | | | 0,77 | | | **0,88** | | |
| **Time** | 266,74 | | | 263,18 | | | 292,90 | | | 319,61 | | | 300,75 | | | **255,09** | | | 265,59 | | |

TP: True Positive; FP: False Positive; TN: True Negative; FN: False Negative; AUC: Area Under the Curve. 0: adenomatoid odontogenic tumour; 1: ameloblastoma; 2: ameloblastic carcinoma.
