# Supplementary figures and images for: Impact of Transfer Learning on Convolutional Neural Networks for Odontogenic Tumor Diagnosis
Source: Head Neck Pathol. 2026 Feb 19;20(1):24. doi: 10.1007/s12105-025-01875-y (PMC12920825; doi:10.1007/s12105-025-01875-y)

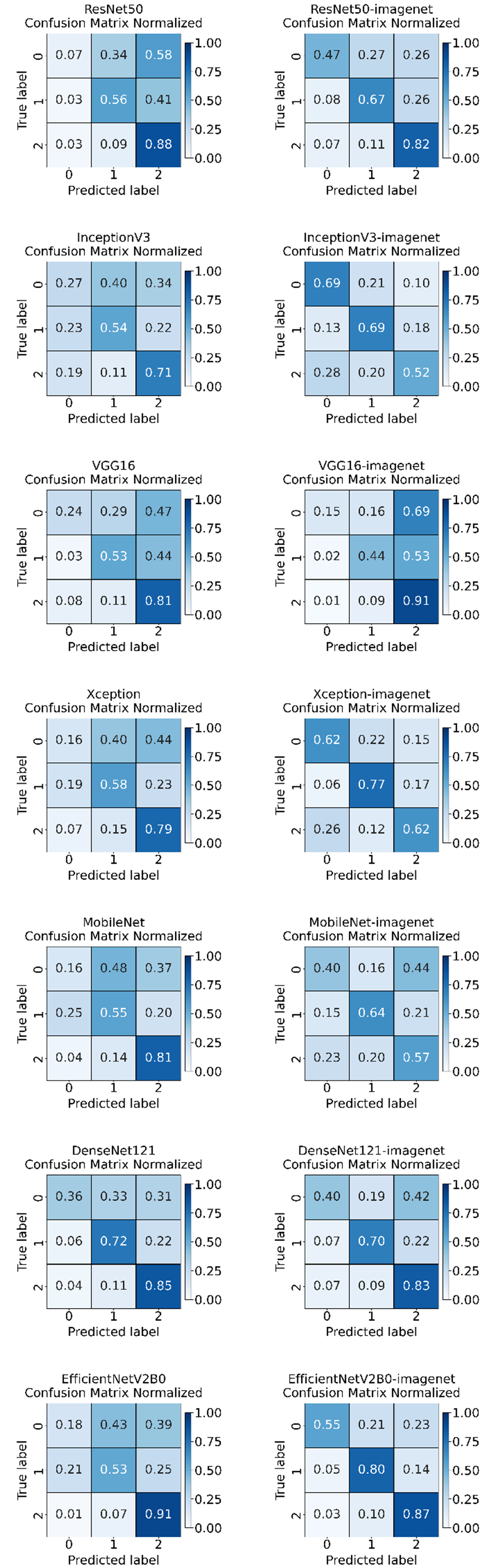

Supplement: Supplementary file 3 — Supplementary Material 3 [file 12105_2025_1875_MOESM3_ESM.tif]

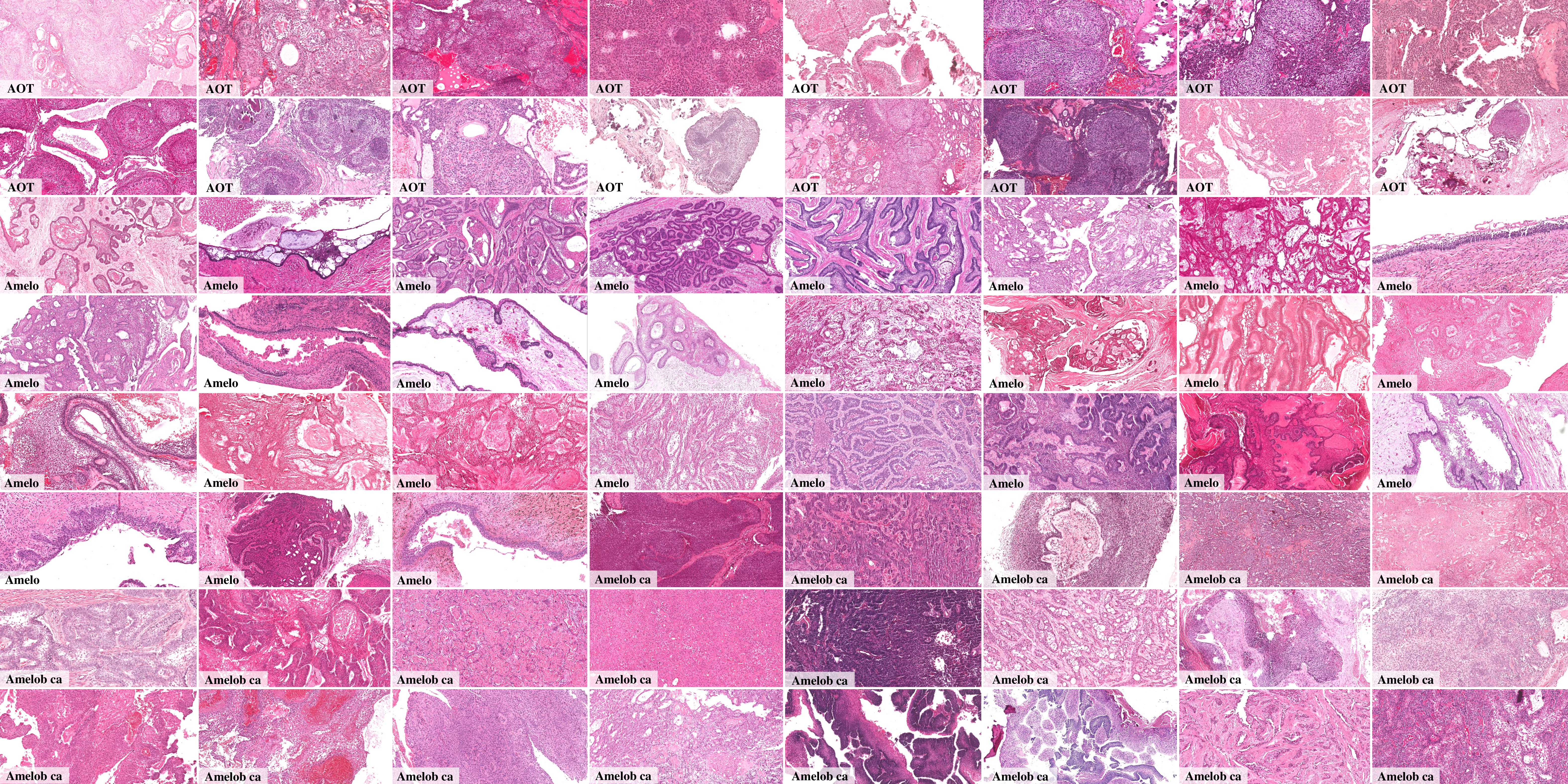

Supplement: Supplementary file 4 — Supplementary Material 4 [file 12105_2025_1875_MOESM4_ESM.tiff]
